# THE BLUES AND OLDER MINORITY MUSICIANS: MORE THAN JUST MUSIC XXIX

**DOI:** 10.1093/geroni/igac059.1591

**Published:** 2022-12-20

**Authors:** John Migliaccio

**Affiliations:** Maturity Mark Services Co., LLC, White Plains, New York, United States

## Abstract

The Blues and Older Minority Musicians: More Than Just Music XXIXThe “ ’Bo Diddley’ Track”; GSA, 2022 Indianapolis, INLecture/Interview/ Performance: TBAIndianapolis has been described as “The Crossroads of America,” with a long history of fostering the music and entrepreneurial spirit of Black Americans. Starting in 1915 with Mrs. C.J. Walker, America’s first female millionaire and her cosmetic enterprise, and the Starr Piano Company and its Gennett Records studio – identified as “The Cradle of Recorded Jazz” in the 1920s and 30s – both were instrumental in national distribution of the works of the earliest blues, jazz, country, and gospel artists including Louis Armstrong, King Oliver, Alberta Hunter, and Charlie Patton among others.Indianapolis continues to host a thriving Blues music community, and will host the 29th consecutive year of GSA’s “Bo Diddley Track” with local older minority musicians, one of GSA’s most popular and fun events. This year will feature a lecture, interview, and mini-performance with leading local blues musicians, followed by a typically raucous live blues performance that evening at a local blues hotspot, with prizes for the first 50 GSA attendees. This has been going on for 29 years for a reason folks---don’t miss it!

